# Guaiacol suppresses osteoclastogenesis by blocking interactions of RANK with TRAF6 and C‐Src and inhibiting NF‐κB, MAPK and AKT pathways

**DOI:** 10.1111/jcmm.15153

**Published:** 2020-03-17

**Authors:** Xin Zhi, Chao Fang, Yanqiu Gu, Huiwen Chen, Xiaofei Chen, Jin Cui, Yan Hu, Weizong Weng, Qirong Zhou, Yajun Wang, Yao Wang, Hao Jiang, Xiaoqun Li, Liehu Cao, Xiao Chen, Jiacan Su

**Affiliations:** ^1^ Department of Orthopedics Trauma Shanghai Changhai Hospital Naval Military Medical University Shanghai China; ^2^ Basic Medical School Naval Military Medical University Shanghai China; ^3^ Department of Pharmacy Shanghai 9th People’s Hospital Shanghai China; ^4^ School of Pharmacy Naval Military Medical University Shanghai China; ^5^ Department of Orthopedics Trauma Shanghai Luodian Hospital Shanghai China; ^6^ Department of Chemistry Fudan University Shanghai China; ^7^ China‐South Korea Bioengineering Center Shanghai China

**Keywords:** guaiacol, osteoclastogenesis, osteoporosis, rankl

## Abstract

Angelica sinensis (AS; Dang Gui), a traditional Chinese herb, has for centuries been used for the treatment of bone diseases, including osteoporosis and osteonecrosis. However, the effective ingredient and underlying mechanisms remain elusive. Here, we identified guaiacol as the active component of AS by two‐dimensional cell membrane chromatography/C18 column/time‐of‐flight mass spectrometry (2D CMC/C18 column/TOFMS). Guaiacol suppressed osteoclastogenesis and osteoclast function in bone marrow monocytes (BMMCs) and RAW264.7 cells in vitro in a dose‐dependent manner. Co‐immunoprecipitation indicated that guaiacol blocked RANK‐TRAF6 association and RANK‐C‐Src association. Moreover, guaiacol prevented phosphorylation of p65, p50, IκB (NF‐κB pathway), ERK, JNK, c‐fos, p38 (MAPK pathway) and Akt (AKT pathway), and reduced the expression levels of Cathepsin K, CTR, MMP‐9 and TRAP. Guaiacol also suppressed the expression of nuclear factor of activated T‐cells cytoplasmic 1(NFATc1) and the RANKL‐induced Ca^2+^ oscillation. In vivo, it ameliorated ovariectomy‐induced bone loss by suppressing excessive osteoclastogenesis. Taken together, our findings suggest that guaiacol inhibits RANKL‐induced osteoclastogenesis by blocking the interactions of RANK with TRAF6 and C‐Src, and by suppressing the NF‐κB, MAPK and AKT signalling pathways. Therefore, this compound shows therapeutic potential for osteoclastogenesis‐related bone diseases, including postmenopausal osteoporosis.

## 1 INTRODUCTION

Angelica sinensis (AS; Dang Gui), a traditional Chinese medicine, has long been used for the treatment of bone diseases including osteoporosis and osteonecrosis.^1^, ^2^, ^3^ In traditional Chinese medicine, AS is considered to increase bone formation and promote fracture healing.^4^ In addition, it reportedly suppresses RANKL‐induced osteoclastogenesis,^5^, ^6^ osteoporosis^7^ and osteonecrosis of the femoral head.^1^ However, the active component and mechanisms underlying the benefits of AS are unclear.

We employed two‐dimensional cell membrane chromatography/C18 column/time‐of‐flight mass spectrometry (2D CMC/C18 column/TOFMS) using a cell membrane immobilized on a silica carrier in the stationary phase. The system facilitates the identification of the active components of herbs^8^, ^9^ and clarifies drug‐receptor interactions.^10^, ^11^ We reported previously that AS‐derived guaiacol has a high affinity for the membrane of bone marrow monocytes (BMMCs).^12^, ^13^

Bone is a dynamic organ modulated by bone formation and resorption.^14^, ^15^ Excess osteoclastogenesis and osteoclast function are closely related to bone loss diseases, such as postmenopausal osteoporosis (PMOP),^16^, ^17^ for which inhibiting osteoclastogenesis and osteoclast function is an important therapeutic strategy.^18^ Osteoclasts are of the monocyte‐macrophage lineage. RANKL and macrophage colony‐stimulating factor (M‐CSF) induce the differentiation of BMMCs into osteoclasts.^14^ RANKL induces activation of the NF‐κB, MAPK and AKT pathways, which is crucial for osteoclastogenesis.^16^ The binding of RANKL to RANK triggers interactions between TRAF6 with RANK,^19^, ^20^ which induces the phosphorylation of p65, p50, IκB (NF‐κB pathway), ERK, JNK, c‐fos and p38 (MAPK pathway). Interactions between RANK and C‐Src result in Akt phosphorylation and activation of the AKT pathway, triggering release of intracellular Ca^2+^ and activation of NFATc1, the master control factor for osteoclastogenesis.^21^, ^22^

Various pharmacological activities of guaiacol have been reported^23^, ^24^; however, its effect on osteoclastogenesis is unknown. Therefore, we evaluated the effects of guaiacol on osteoclastogenesis in vivo and in vitro.

## 2 MATERIALS AND METHODS

### 2.1 Reagents

Guaiacol was provided by the Standard Company. RAW264.7 cells were supplied by the Chinese Academy of Sciences Cell Institute (Shanghai, China). Foetal bovine serum (FBS), alpha‐modified minimal essential medium (α‐MEM), penicillin and streptomycin were obtained from Hyclone. The Cell Counting Kit‐8 (CCK‐8), tartrate‐resistant acid phosphatase (TRAP), alkaline phosphatase (ALP), alizarin red and oil red O staining kits were purchased from Sigma‐Aldrich. Recombinant soluble M‐CSF and RANKL were provided by R&D Systems.

### 2.2 Identification of active components

Bone marrow monocytes were combined with AS (Dang Gui) and 2D BMMCs/CMC/C18 column/TOFMS analyses were carried out to assess the affinity of components of AS for the membrane of BMMCs as reported previously.^12^, ^25^ Cell membrane chromatography (CMC) is a biological affinity chromatographic technique in which specific cell membrane contained certain receptors as stationary phase. In brief, by immersing silica into a suspension of cell membranes, the whole surface of silica was covered by the cell membranes due to the irreversible adsorption of silanol groups (Si−OH) on the silica surface and the self‐fusion of the cell membranes, which can be used directly as a chromatographic stationary phase, and then packed into a column to build a CMC model. The retention time of compounds on CMC columns depends on the affinity between compounds and receptors on membrane. The stronger the affinity, the longer the retention time is. Retention time of compounds was recorded by Agilent MassHunter Workstation (Agilent Technologies) and a home‐written program in Visual Basic 6.0 (Microsoft). Therefore, CMC can realize high‐throughput screening of active components of traditional Chinese medicines. Compounds with long retention time can be considered as potential active ingredients. The screened active components were identified based on the Traditional Chinese Medicine and Chemical Composition Database (http://202.127.145.134/scdb/main/tcm_introduce.asp).

### 2.3 Ovariectomy‐induced in vivo model and experimental procedure

Female C57BL/6 mice (8 weeks old) were obtained from Weitonglihua Company. The mice were maintained, and animal experiments were conducted, in a specific pathogen‐free laboratory (Changhai Hospital). The experimental protocols conformed to the standards of the Bioethics Committee of Changhai Hospital (SYXK 2015‐0017) and that institution's guidelines for the care and use of animals. Following intraperitoneal anaesthesia, OVX‐induced mice were generated by removing the bilateral ovaries, followed by recovery for 1 day. Mice were randomly assigned to the following three groups (n = 6/group): the sham‐operated, OVX (OVX mice treated with normal saline) and guaiacol groups (OVX mice treated with guaiacol). Over the following 6 weeks, mice in the OVX and guaiacol groups were intraperitoneally administered normal saline and guaiacol (125 mg/kg) daily, respectively. The mice were euthanized, and the bilateral femurs and arterial blood were removed.

### 2.4 CCK‐8 assay

Bone marrow monocytes and RAW264.7 cells were cultured in a 96‐well plate at 1 × 10^4^/well (100 μL) per well and treated with guaiacol at the indicated concentrations for 48 hours. Next, 10 µL CCK‐8 solution (R&D Systems) was added, and the cells were cultured for a further 2 hours. Finally, an enzyme‐linked immunosorbent assay (ELISA) plate reader was employed to measure the absorbance at 450 nm to evaluate the cytotoxicity of guaiacol. All experiments were conducted for three times, and the average was calculated.

### 2.5 Osteoclastogenesis assay

Bone marrow monocytes were extracted from the marrow of mouse femurs, and RAW264.7 cells and BMMCs were cultured in α‐MEM containing 1% penicillin, 1% streptomycin and 10% FBS and maintained in an incubator. Next, the cells (1 × 10^4^/well) were seeded into 96‐well plates containing M‐CSF (30 ng/mL) and RANKL (50 ng/mL) in the presence or absence of guaiacol (0.25, 0.5 or 1.0 μmol/L). The cells were subjected to TRAP staining on day 7. All experiments were conducted for 3 times, and the average was calculated.

### 2.6 Bone resorption assay

RAW264.7 cells (1 × 10^4^/well) were induced with M‐CSF (30 ng/mL) and RANKL (50 ng/mL) in a culture plate. After osteoclast induction and collagenase digestion, cells were seeded on the bone biomimetic synthetic surface (Corning) and incubated with guaiacol (0.25, 0.5, and 1.0 μmol/L) and 30 ng/mL M‐CSF and 50 ng/mL RANKL for 2 days. The culture medium was replaced on day 3. The cells were washed in PBS and dried for 3 hours. The resorbed area was visualized under a microscope (Olympus BX53) and quantified using Image J software. All experiments were conducted for three times, and the average was calculated.

### 2.7 Induction of osteogenesis and adipogenesis

Bone marrow mesenchymal stem cells (BMSCs) were extracted from the marrow of mouse femurs. The cells were incubated in complete medium containing 10 mmol/L β‐glycerophosphate, 0.1 μmol/L dexamethasone (DXM), 5 μg/mL insulin and 0.2 mmol/L ascorbic acid to stimulate osteogenesis. Osteoblast differentiation was visualized by ALP staining, and the mineral nodes of mature osteoblasts were visualized by alizarin red staining. Adipogenesis was induced by culturing the BMSCs in complete medium containing 10 μg/mL insulin, 0.1 mmol/L indomethacin, 1.0 μmol/L DXM and 0.5 mmol/L 3‐isobutyl‐1‐methyl xanthine. Finally, differentiated cells were stained with oil red O. All experiments were conducted for 3 times, and the average was calculated.

### 2.8 Microcomputed tomography

The distal femur metaphysis was subjected to microcomputed tomography (micro‐CT; Skyscan) at 8 μmol/L resolution, 80 kV X‐ray voltage and 124 μA current. The following structural parameters were analysed in scanned images: trabecular number, bone volume/total volume, bone surface area/total volume and bone mineral density. Two‐ and three‐dimensional images of the femur structure were reconstructed.

### 2.9 Histologic analyses

Mouse femurs were fixed in paraformaldehyde (PFA; 4% Sigma‐Aldrich) for 4 days. After decalcification for 2 weeks in 10% ethylenediaminetetraacetic acid (Solarbio), the femurs were paraffin‐embedded and sectioned at 4 μmol/L thickness. Thereafter, the sections were stained with haematoxylin and eosin (H&E), OCN, and TRAP (Sigma‐Aldrich). The femur trabecular area was visualized under a microscope (Olympus BX53); TRAP‐positive cells with three or more nuclei were considered mature osteoclasts.

### 2.10 Serum biochemistry detection

Blood was removed from the mice by ophthalmocentesis, centrifuged at 1000 *g* for 20 minutes, and then serum was extracted. Serum levels CTX‐1 and TRAcp5B were measured using an ELISA kit (Anogen) in accordance with the company's protocols.

### 2.11 Immunofluorescence staining of p65, F‐actin rings and NFATc1

RAW264.7 cells were stimulated with M‐CSF (30 ng/mL) and RANKL (50 ng/mL) with various concentrations of guaiacol. After fixation with 4% PFA and washing in PBS, cells were permeabilized with 0.1% TritonX and blocked in 3% bovine serum albumin. Nuclei were stained with 4,′6‐diamidino‐2‐phenylindole (Sigma), and the cells were reacted with anti‐p65, anti‐F‐actin, and anti‐NFATc1 antibodies. Next, the cells were cultured with fluorescein isothiocyanate‐ and cyanine 3‐conjugated secondary antibodies for 1 hour, counterstained with propidium iodide and visualized via confocal laser scanning microscopy (Olympus). All experiments were conducted for three times, and the average was calculated.

### 2.12 Measurement of intracellular Ca^2+^ levels

Bone marrow monocytes were cultured in 96‐well plates (1 × 10^4^/well) with M‐CSF (30 ng/mL) and RANKL (50 ng/mL) in the presence or absence of guaiacol (0.25, 0.5, and 1.0 μmol/L). Briefly, after washing with assay buffer, 4 μmol/L Fluo4 staining solution was added to the cells. Intracellular Ca^2+^ was visualized using an inverted fluorescence microscope (Nikon Ti‐U) at 488 nm, together with Nikon Basic Research Software. Images were scanned at 2 seconds intervals for 3 minutes. Cells with two or more peaks were considered oscillating. We recorded the difference between the highest and lowest fluorescence intensities in the area of oscillation. All experiments were conducted for 3 times, and the average was calculated.

### 2.13 Quantitative real‐time PCR

Total RNA was isolated using TRIzol reagent (Invitrogen), and cDNA was reverse transcribed from the RNA (Invitrogen). RT‐PCR was performed using an ABI ViiA7 Real‐Time System (Applied Biosystems) with the following primers: RANK forward (5′‐CTGCTCCTCTTCATCTCTGTG‐3′), RANK reverse (5′‐CTTCTGGAACCATCTTCTCCTC‐3′), C‐Fms forward (5′‐TTCACTCCGGTGGTGGTGGCCTGT‐3′) and C‐Fms reverse (5′‐GTTGAGTAGGTCTCCATAGCAGCA‐3′). All experiments were conducted for three times, and the average was calculated.

### 2.14 Western blotting

Western blotting was performed to examine the phosphorylation of p50, p65, IκB (NF‐κB pathway), Akt (AKT pathway), p38, ERK, C‐fos and JNK (MAPK pathway) in RAW264.7 cells. Cells induced by M‐CSF (30 ng/mL) and RANKL (50 ng/mL) with or without guaiacol (0 and 1.0 μmol/L) were incubated in a 96‐well plate for 7 days. Next, the expression levels of osteoclastogenesis‐related genes (encoding cathepsin K, CTR, MMP‐9 and TRAP) were assayed. Proteins were prepared and quantified using a bicinchoninic acid (BCA) kit (Thermo Fisher), resolved by sodium dodecyl sulphate‐polyacrylamide gel electrophoresis, electrotransferred onto a membrane, and blocked in Tris‐buffered saline with Tween in 5% skim milk. After incubation with the primary antibodies overnight (4°C), the samples were incubated with anti‐rabbit horseradish peroxidase‐conjugated secondary antibodies. The results were visualized by chemiluminescence (Bio‐Rad). All experiments were conducted for 3 times, and the average was calculated.

### 2.15 Co‐immunoprecipitation assay

After lysis and centrifugation, the supernatant of RAW264.7 cells was added to TRAF6 or C‐Src and the corresponding specific IgG. The mixtures were cultured with IgG agarose beads, and the results were visualized by Western blotting. All experiments were conducted for three times, and the average was calculated.

### 2.16 Statistical analyses

Data are means ± standard deviation (SDs) of triplicate assays and were analysed using SPSS ver. 20.0 software. Comparisons of two groups were performed using two‐tailed, unpaired Student's *t* test. Comparisons of three or more groups were performed using one‐way analysis of variance.

## 3 RESULTS

### 3.1 Guaiacol is the active component of AS

**Figure 1:**
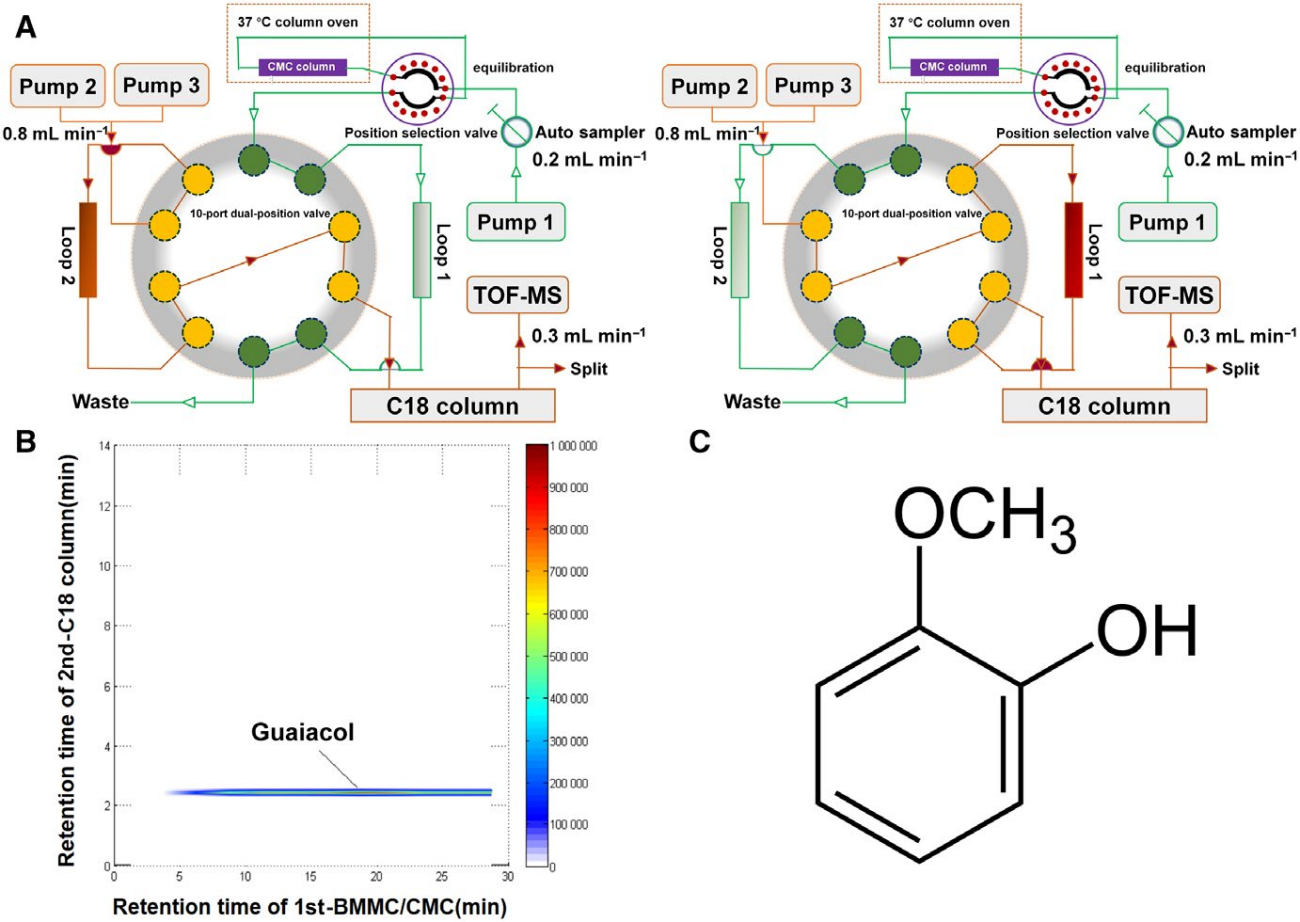
Guaiacol extracted from AS. A, The 2D CMC/C18 column/TOFMS system. B, Typical 2D chromatograph of guaiacol. C, Molecular formula of guaiacol

BMMCs/CMC/C18 column/TOFMS analyses (Figure 1A) showed that there was good affinity between a component of AS and the membrane of BMMCs. This component had strong retention behaviour, with a peak at 20 minutes (Figure 1B), suggesting that it could combine with the BMMC membrane and possibly inhibit osteoclastogenesis. No other component interacted with the membrane. The molecular formula of the active component was C_7_H_8_O_2_, and comparison with known compounds of AS using the Traditional Chinese Medicine Integrated Database resulted in its identification as guaiacol (Figure 1C).

### 3.2 Guaiacol suppresses osteoclastogenesis in vitro

**Figure 2:**
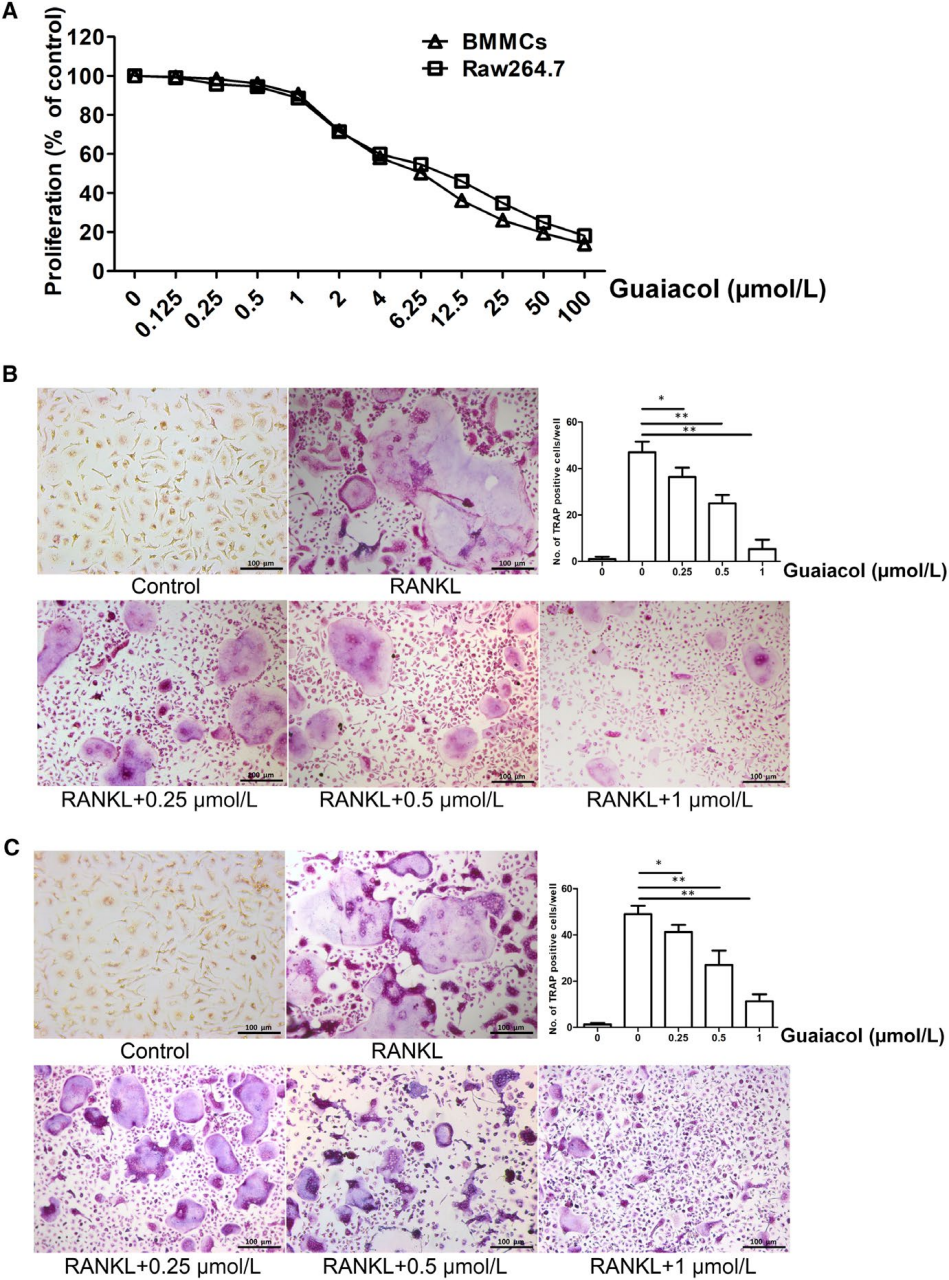
Guaiacol suppressed osteoclastogenesis in vitro. A, CCK‐8 assay of guaiacol cytotoxicity against BMMCs and RAW264.7 cells. B, TRAP‐positive cells induced from BMMCs and number of osteoclasts. C, TRAP‐positive cells induced from RAW264.7 cells and number of osteoclasts. Induced BMMCs and RAW264.7 cells were treated with 0.25, 0.5 and 1.0 μmol/L guaiacol. *P < .05, **P < .01

The results of CCK‐8 assays indicated that guaiacol at <1.0 µmol/L showed little cytotoxicity (Figure 2A). TRAP assays indicated that M‐CSF and RANKL induced osteoclast differentiation, which was strongly suppressed by guaiacol in a dose‐dependent manner (Figure 2B,C). Therefore, guaiacol inhibits osteoclastogenesis.

### 3.3 Guaiacol inhibits osteoclast function

**Figure 3:**
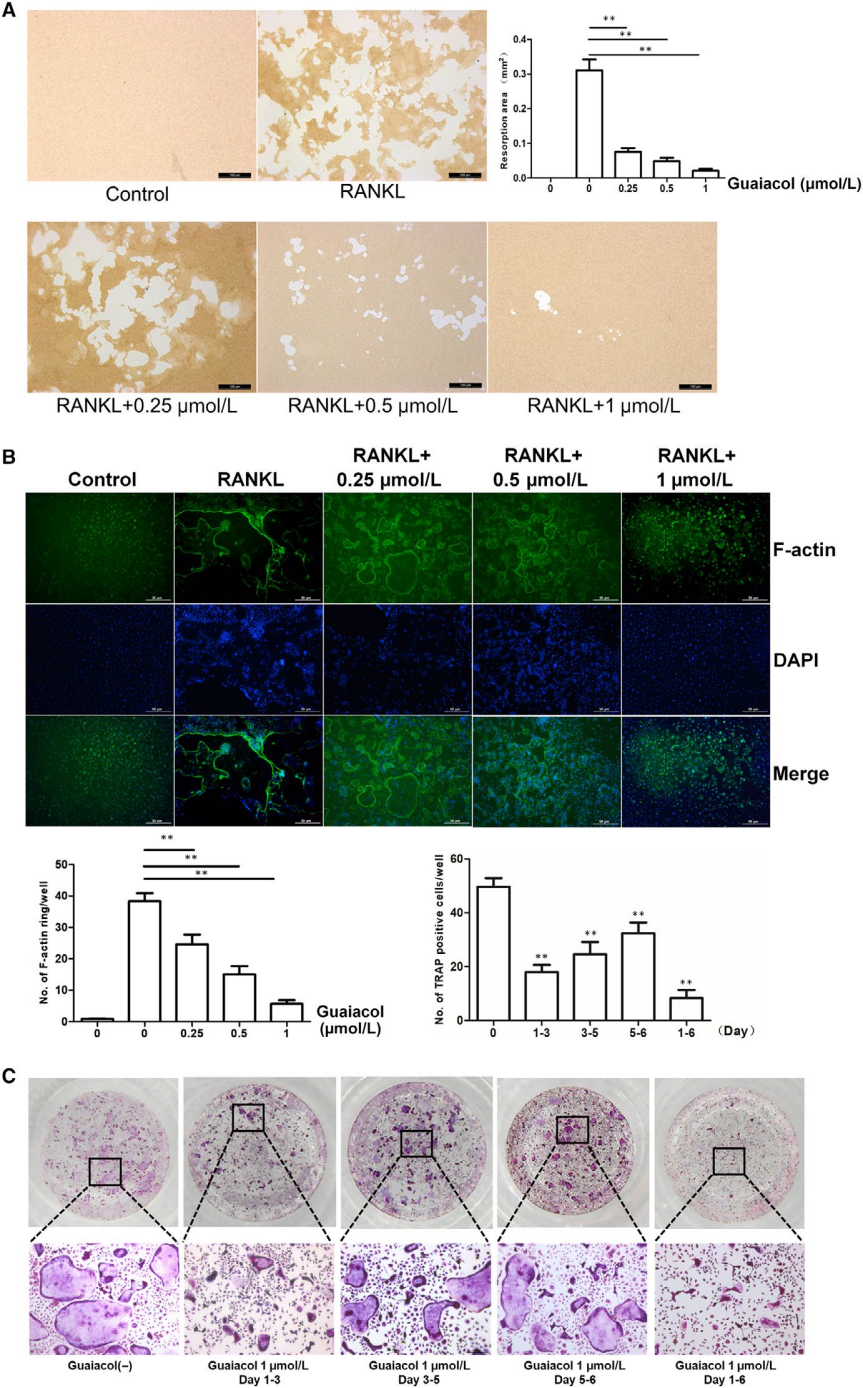
Guaiacol inhibited osteoclast function and attenuated early osteoclastogenesis. A, Pit‐forming assay of osteoclasts and quantification of pit area. B, F‐actin ring formation by osteoclasts and quantification of actin rings. RAW264.7 cells were stimulated with M‐CSF and RANKL and treated with 0.25, 0.5 and 1.0 μmol/L guaiacol. C, Effect of 1.0 μmol/L guaiacol on the number of TRAP‐positive cells. **P < .01

A pit‐formation assay using the bone biomimetic synthetic surface showed that the resorbed area was dose‐dependently diminished by guaiacol (Figure 3A). Immunofluorescence staining demonstrated that the formation of f‐actin ring structures was markedly suppressed by guaiacol (Figure 3B). Therefore, guaiacol inhibits osteoclast function.

### 3.4 Guaiacol attenuates early osteoclastogenesis

To determine the stage of osteoclastogenesis inhibited by guaiacol, BMMCs were induced by M‐CSF and RANKL, and treated with guaiacol. Guaiacol suppressed RANKL‐induced osteoclast differentiation in the first few days; however, the effect decreased in magnitude thereafter (Figure 3C). Therefore, guaiacol primarily suppresses early‐stage osteoclast differentiation.

### 3.5 Guaiacol does not affect the proliferation of BMMCs

During osteoclast formation, M‐CSF induces BMMCs to differentiate into pre‐osteoclasts, which fuse to multinucleated osteoclasts inducted by RANKL. Hence, we examined the impact of guaiacol on the proliferation of BMMCs. CCK‐8 assays showed that M‐CSF‐induced proliferation of BMMCs was not significantly affected by <1.0 μmol/L guaiacol (Figure S1). Therefore, guaiacol does not inhibit the proliferation of BMMCs.

### 3.6 Guaiacol does not influence RANK or c‐Fms expression during osteoclastogenesis

To assess the effects of guaiacol on receptor of RANKL (RANK) and M‐CSF (c‐Fms), the expression levels of RANK and c‐Fms were assayed via RT‐PCR. The expression of c‐Fms was unaffected by stimulation with M‐CSF or guaiacol. Induction by M‐CSF increased the expression of RANK compared with controls. In addition, RANK expression was not affected by guaiacol (Figure S2). Similarly, immunofluorescence analyses showed that the expression of RANK and c‐Fms was not influenced by guaiacol (Figure S3). Therefore, guaiacol did not affect the expression of RANK and c‐Fms during osteoclastogenesis.

### 3.7 Guaiacol does not influence osteogenesis and adipogenesis by BMSCs

We evaluated the effects of guaiacol on osteogenesis and adipogenesis by BMSCs using oil red O, ALP and alizarin red staining. Osteogenesis (Figure S4) and adipogenesis (Figure S5) were not significantly inhibited by guaiacol. Therefore, guaiacol predominately functions as an osteoclastogenesis inhibitor.

### 3.8 Guaiacol inhibits the RANKL‐induced activation of the NF‐κB and MAPK pathways and blocks the RANK‐TRAF6 interaction

**Figure 4:**
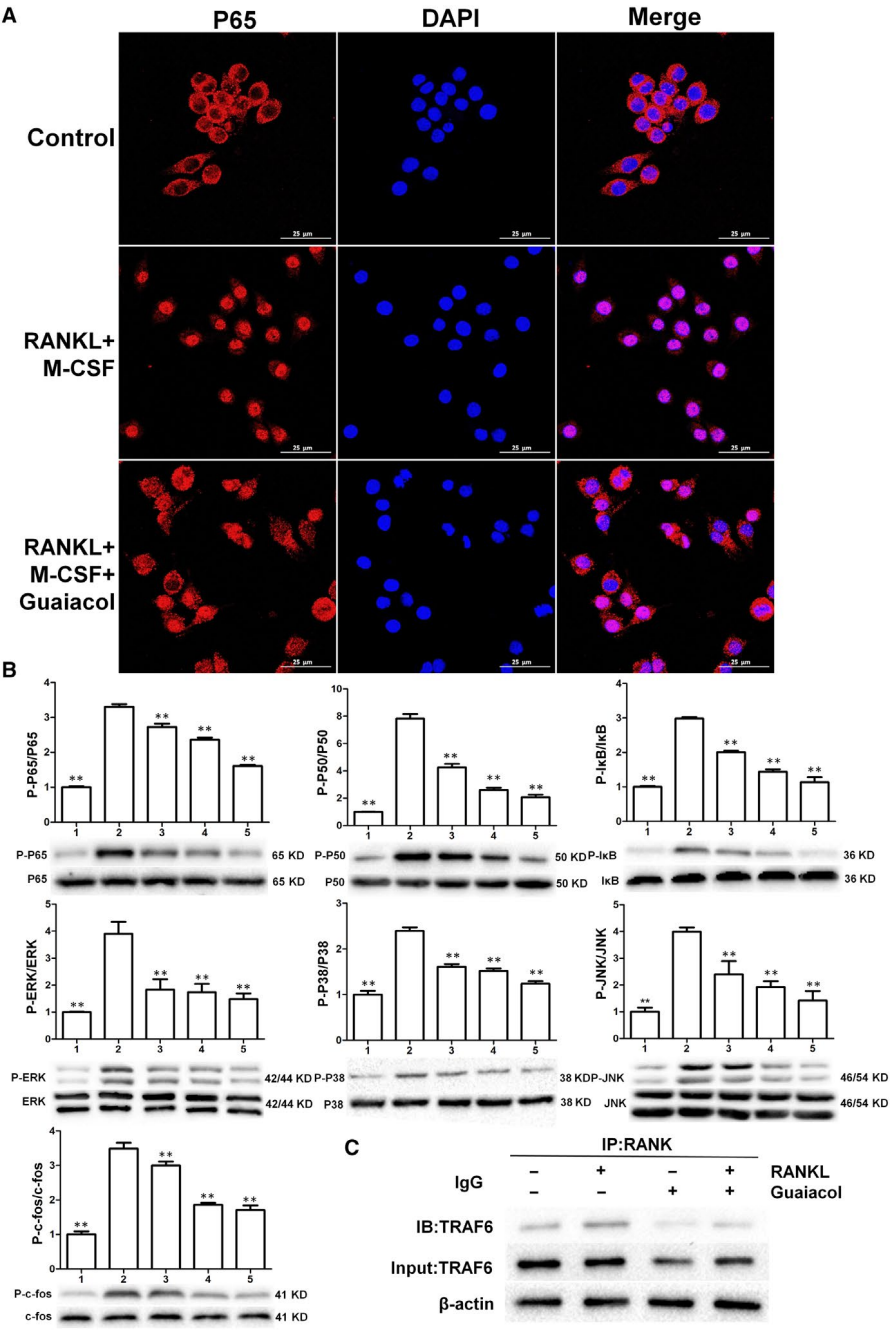
Guaiacol inhibited RANKL‐induced activation of the NF‐κB and MAPK pathways and blocked the RANK‐TRAF6 interaction. A, Guaiacol suppressed RANKL‐mediated nuclear translocation of p65. B, Phosphorylation levels of components of the NF‐κB (p50, p65 and IκB) and MAPK (ERK, JNK, C‐fos and p38) pathways. C, Guaiacol suppressed the RANK‐TRAF6 interaction. 1. RAW264.7 cells; 2. RAW264.7 cells induced with M‐CSF (30 ng/mL) and RANKL (50 ng/mL), and PBS; 3. RAW264.7 cells induced with M‐CSF (30 ng/mL) and RANKL (50 ng/mL) and treated with 0.25 μmol/L guaiacol; 4. RAW264.7 cells induced with M‐CSF (30 ng/mL) and RANKL (50 ng/mL) and treated with 0.5 μmol/L guaiacol; 5. RAW264.7 cells induced with M‐CSF (30 ng/mL) and RANKL (50 ng/mL) and treated with 1 μmol/L guaiacol. **P < .01

RANKL‐induced activation of the NF‐κB and MAPK pathways is essential for osteoclastogenesis. p65, a downstream factor of the NF‐κB pathway, is normally present in the cytoplasm. We performed immunofluorescence staining for p65 to explore whether guaiacol may suppress NF‐κB‐mediated osteoclastogenesis in RAW264.7 cells. M‐CSF and RANKL stimulation increased the nuclear translocation of p65, which was notably attenuated by guaiacol (Figure 4A). In addition, the phosphorylation of downstream components of the MAPK pathway (ERK, JNK, c‐fos and p38) was decreased by guaiacol (Figure 4B). Therefore, guaiacol significantly suppresses RANKL‐mediated activation of the NF‐κB and MAPK pathways during osteoclastogenesis. Next, we performed co‐IP to determine whether guaiacol may recruit TRAF6 after activation of RANK in RAW264.7 cells. RANKL promoted interactions between RANK and TRAF6; however, guaiacol markedly suppressed this process (Figure 4C). Therefore, the binding of RANKL to RANK stimulates recruitment of TRAF6, which is suppressed by guaiacol.

### 3.9 Guaiacol inhibits RANKL‐induced activation of the AKT pathway and blocks the RANK‐C‐Src interaction

**Figure 5:**
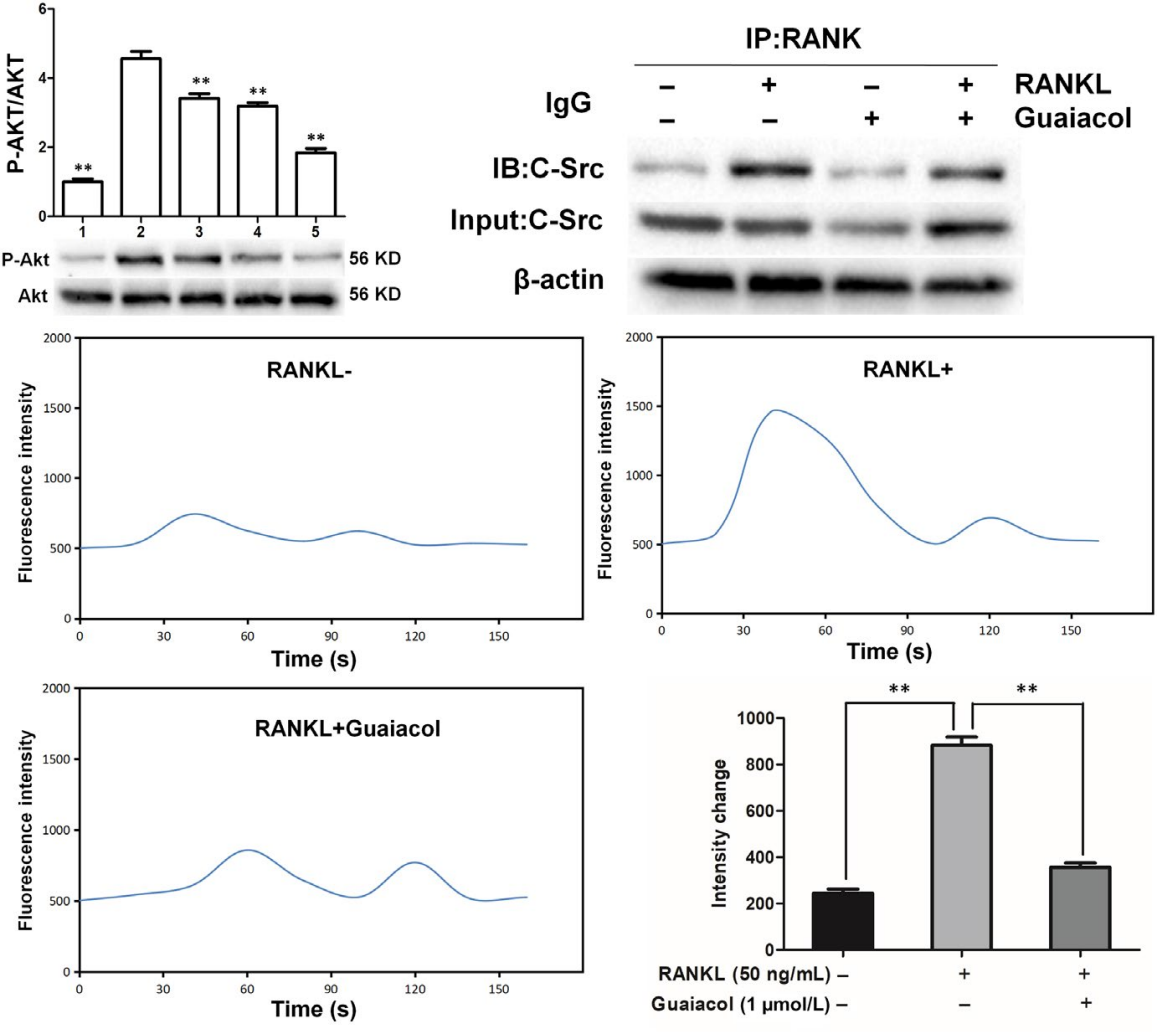
Guaiacol inhibited RANKL‐induced activation of the AKT pathway, blocked the RANK‐C‐Src interaction and attenuated release of intracellular Ca^2+^ during osteoclastogenesis. A, Phosphorylation levels of components of the AKT pathway (Akt). B, Guaiacol suppressed the RANK‐C‐Src interaction. C, Representative images of Ca^2+^ oscillation in RANKL‐induced BMMCs; guaiacol inhibited the RANKL‐induced Ca^2+^ oscillation. 1. RAW264.7 cells; 2. RAW264.7 cells induced with M‐CSF (30 ng/mL), RANKL (50 ng/mL) and PBS; 3. RAW264.7 cells induced with M‐CSF (30 ng/mL) and RANKL (50 ng/mL) and treated with 0.25 μmol/L guaiacol; 4. RAW264.7 cells induced with M‐CSF (30 ng/mL) and RANKL (50 ng/mL) and treated with 0.5 μmol/L guaiacol; 5. RAW264.7 cells induced with M‐CSF (30 ng/mL) and RANKL (50 ng/mL) and treated with 1 μmol/L guaiacol. **P < .01

Stimulation of the AKT pathway is vital for osteoclast differentiation and function. We investigated whether guaiacol may suppress the RANKL‐induced activation of the AKT pathway. Western blotting showed that the phosphorylation level of Akt was markedly elevated by RANKL but considerably decreased by guaiacol (Figure 5A). Co‐IP showed that RANKL promoted the interaction of RANK with C‐Src, which was significantly inhibited by guaiacol (Figure 5B). Activation of the AKT pathway leads to release of intracellular Ca^2+^. The RANKL‐induced release of intracellular Ca^2+^ was notably inhibited by guaiacol (Figure 5C).

### 3.10 Guaiacol suppresses the expression of NFATc1 and osteoclastogenesis‐related genes during osteoclastogenesis

**Figure 6:**
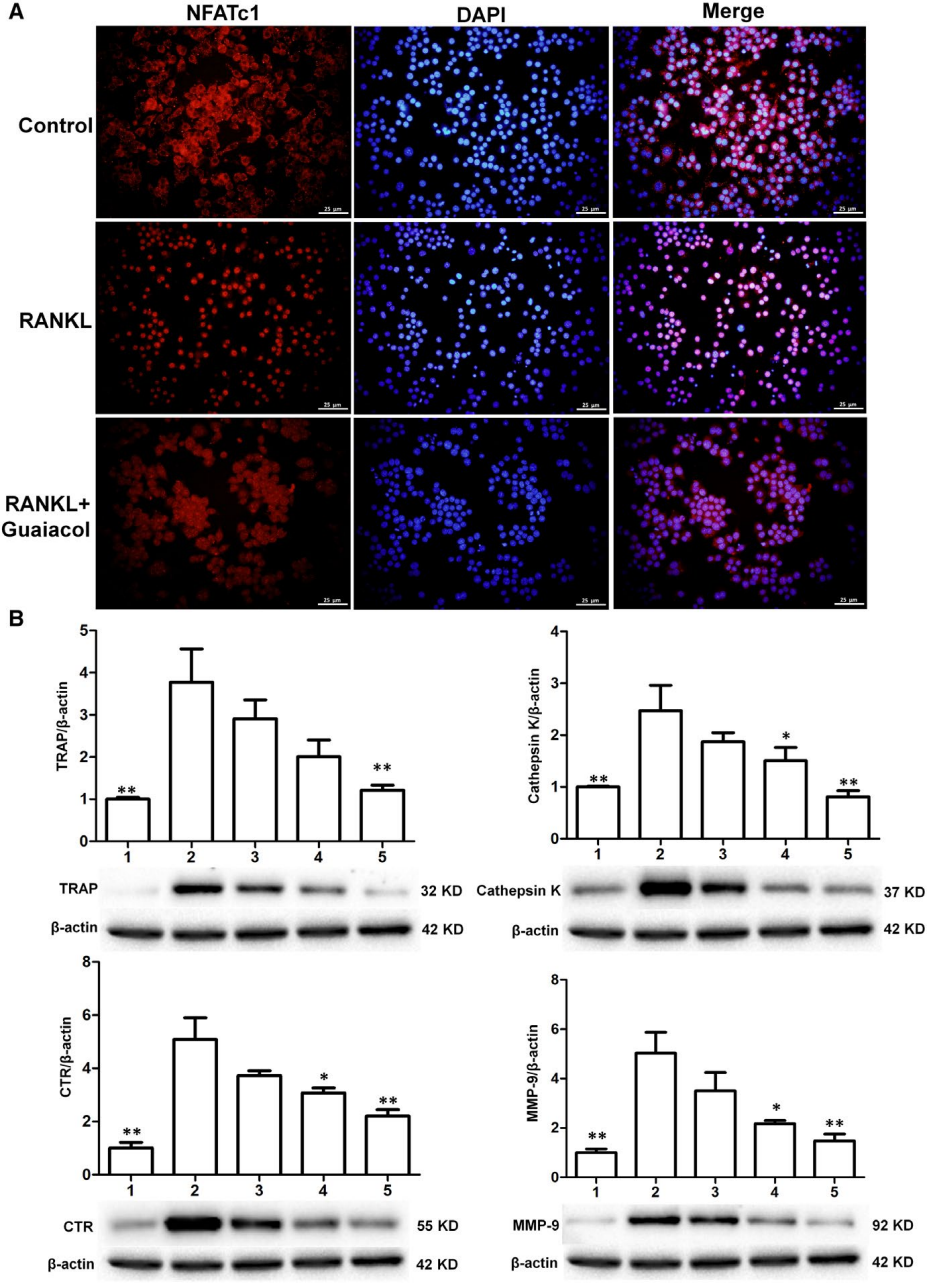
Guaiacol suppressed the expression of NFATc1 and osteoclastogenesis‐related genes. A, RANKL induced the nuclear translocation of NFATc1 during osteoclastogenesis, while guaiacol inhibited NFATc1 expression. B, Western blotting of cathepsin K, CTR, MMP‐9 and TRAP, with β‐actin as a reference. 1. RAW264.7 cells; 2. RAW264.7 cells induced with M‐CSF (30 ng/mL), RANKL (50 ng/mL) and PBS; 3. RAW264.7 cells induced with M‐CSF (30 ng/mL) and RANKL (50 ng/mL) and treated with 0.25 μmol/L guaiacol; 4. RAW264.7 cells induced with M‐CSF (30 ng/mL) and RANKL (50 ng/mL) and treated with 0.5 μmol/L guaiacol; 5. RAW264.7 cells induced with M‐CSF (30 ng/mL) and RANKL (50 ng/mL) and treated with 1 μmol/L guaiacol. *P < .05, **P < .01

NFATc1 modulates the expression of genes associated with osteoclast differentiation and bone resorption. We performed immunofluorescence staining to evaluate the effects of guaiacol on the transcriptional activity of NFATc1. The RANKL‐induced nuclear translocation of NFATc1 was activated during osteoclastogenesis but was decreased by guaiacol (Figure 6A). Several genes are markers of osteoclast activation and differentiation, including those encoding cathepsin K, CTR, MMP‐9 and TRAP. The expression levels of these genes in RAW264.7 cells were significantly diminished by guaiacol in a dose‐dependent manner (Figure 6B). Therefore, guaiacol inhibits osteoclastogenesis and osteoclast bone‐resorbing activity by modulating the expression of osteoclastogenesis‐related genes.

### 3.11 Guaiacol inhibits bone loss in OVX mice

**Figure 7:**
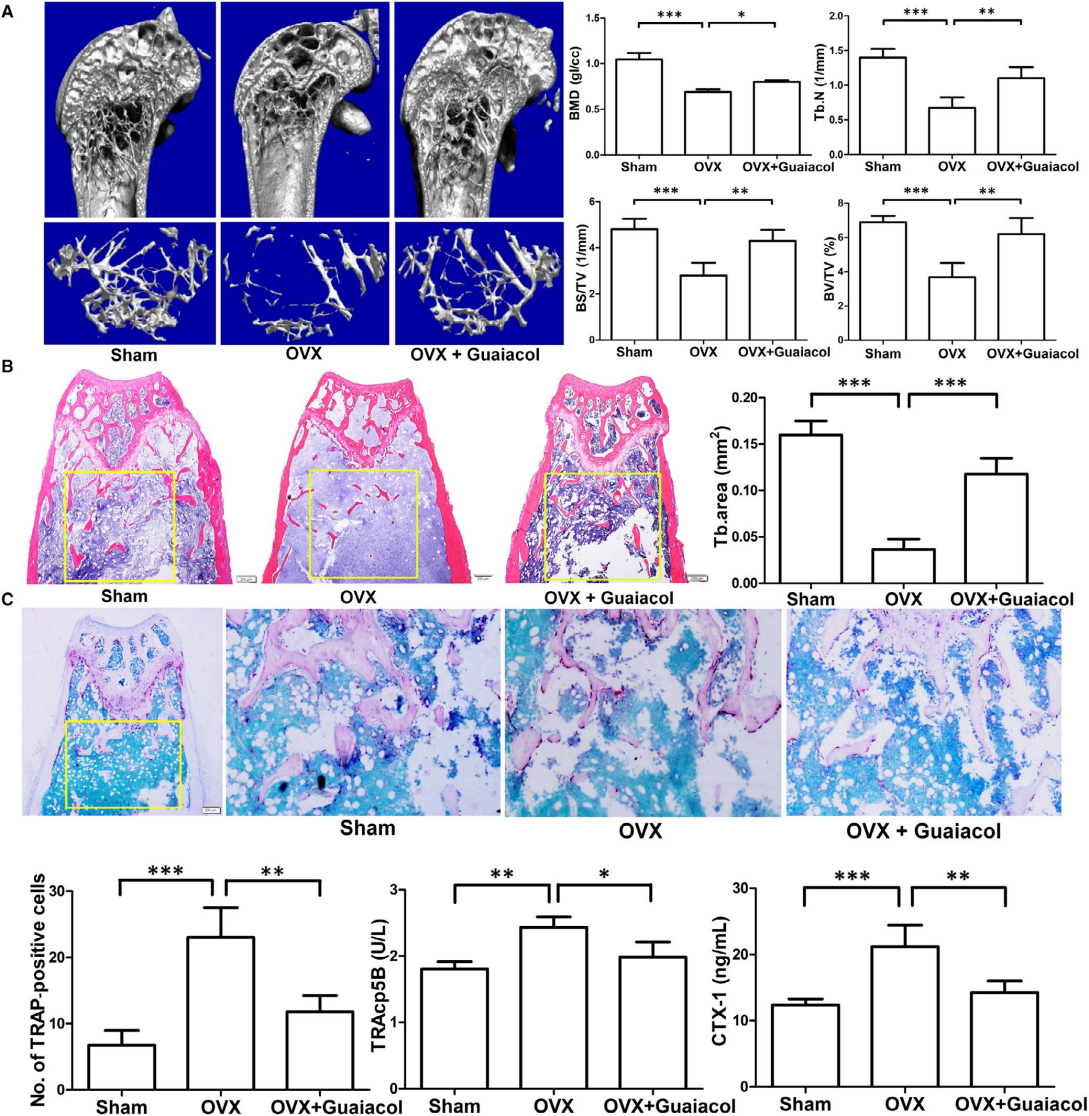
Guaiacol inhibited bone loss in OVX mice. A, Micro‐CT analyses of the distal femur of mice in the sham, OVX, and OVX + guaiacol groups. B, H&E staining of sections of the distal femur and trabecular area at 6 wk after treatment. C, TRAP‐stained sections of the distal femur and number of TRAP‐positive cells in mice in the sham, OVX, and OVX + guaiacol groups. D, Level of TRAcp5B and CTX‐1 as determined by ELISA. *P < .05, **P < .01, ***P < .001

The mice in the OVX group showed marked trabecular bone loss relative to those in the sham group; the degree of loss was markedly rescued by guaiacol (Figure 7A). Similarly, H&E staining showed that the area of trabecular bone was larger in the guaiacol group than in the OVX group (Figure 7B). In addition, compared with the OVX group, TRAP staining showed that guaiacol decreased the number of mature osteoclasts (Figure 7C). In addition, the serum levels of CTX‐1 and TRAcp5B were lower in the guaiacol group than in the OVX group, suggesting that osteoclast activity was significantly attenuated by guaiacol (Figure 7D). However, OCN staining and serum levels of OCN suggested that osteogenesis was not affected by guaiacol (Figure S6). Therefore, guaiacol inhibits OVX‐induced bone loss by suppressing osteoclastogenesis in vivo.

## 4 DISCUSSION

**Figure 8:**
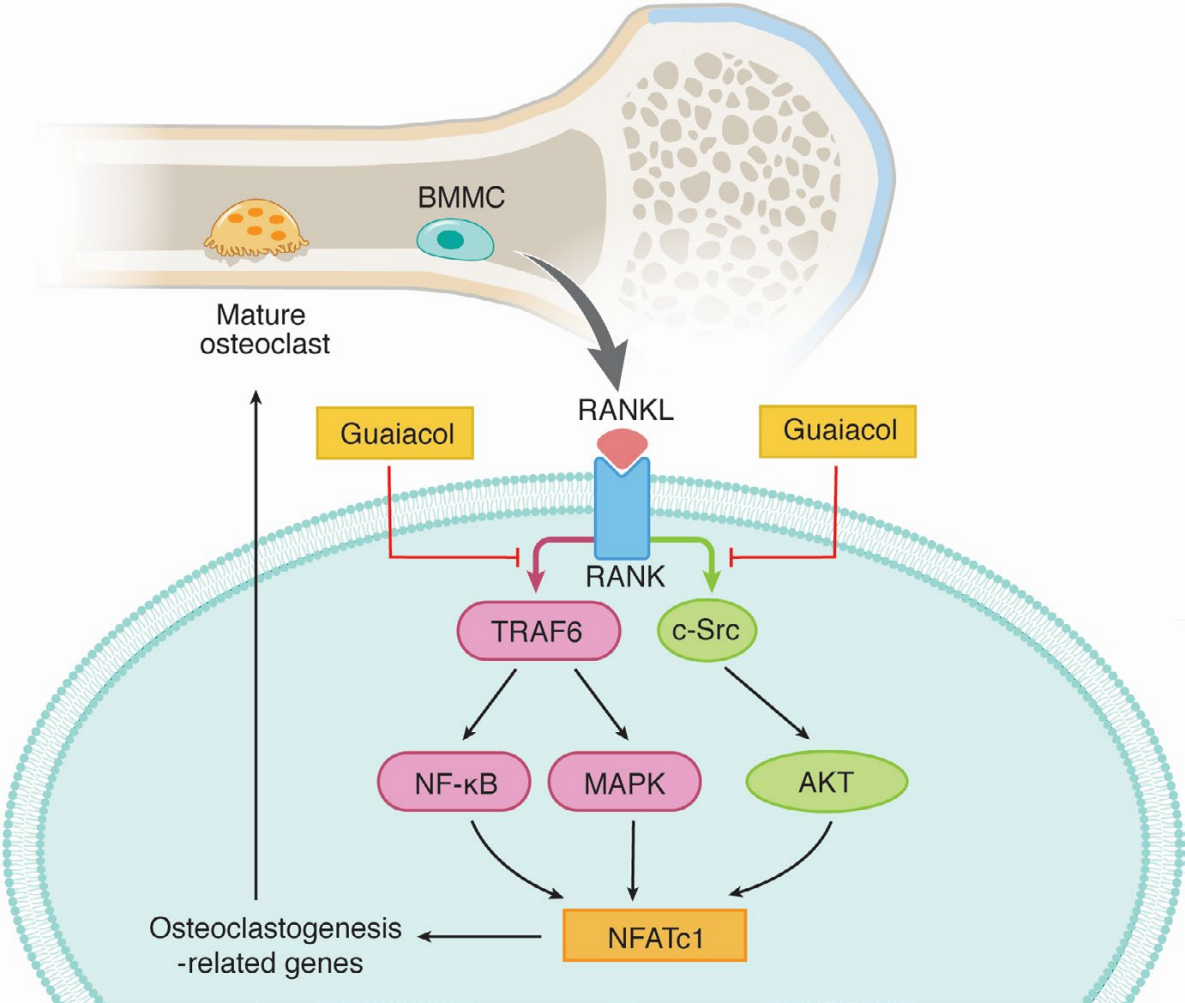
Mechanisms by which guaiacol inhibits osteoclast differentiation and function via multiple signalling pathways

We identified guaiacol as an active component of AS by 2D BMMC/CMC/C18 column/TOFMS analyses and found that it significantly inhibited osteoclast function and osteoclastogenesis in vitro. In addition, it decreased RANKL/M‐CSF‐induced activation of the NF‐κB, MAPK and AKT pathways at the early stage by blocking interactions between RANK and TRAF6 and C‐Src. In vivo, it significantly attenuated OVX‐induced bone loss (Figure 8).

Angelica sinensis, a traditional Chinese medicine, has been widely used to treat bone diseases including bone fractures, osteoporosis and osteonecrosis.^1^, ^2^, ^3^ AS suppresses RANKL‐induced osteoclastogenesis,^5^, ^6^ osteoporosis^7^ and osteonecrosis of the femoral head.^1^ However, the active component and mechanisms underlying its activity were unclear.

We identified the active component (C_7_H_8_O_2_) as guaiacol and found that it inhibited osteoclastogenesis. Employing the 2D BMMCs/CMC/C18 column/TOFMS system, we detected the affinity of AS and BMMCs cell membrane. Finally, C_7_H_8_O_2_ was filtrated and compared with the known ingredients of AS. It was identified as guaiacol and considered could inhibit osteoclastogenesis. This system based on the cell membrane chromatography and utilized the advantages of online high‐throughput processing and biological activity, which was very efficient for the filtration of potential active ingredients from complex system, especially from herbal medicine. This system will be employed to enclosure the active ingredients of Chinese herbal medicine and explore the mechanisms of Chinese medicine in future.

Bone is constantly renewed via bone formation by osteoblasts and bone resorption by osteoclasts. Excessive bone resorption by osteoclasts leads to pathological bone loss. Therefore, inhibition of osteoclastogenesis and osteoclast function is an important therapeutic strategy for diseases caused by bone loss, such as PMOP and arthritis.^26^ Although anti‐bone resorption drugs are used to treat bone loss diseases, they have several side effects and their efficacy is inadequate.^27^ Therefore, a safer and more efficacious agent for the treatment of bone loss diseases is required; the active ingredients of traditional Chinese medicines could meet this need.

We found that <1.0 µmol/L guaiacol had little cytotoxic effect on RAW264.7 cells and BMMCs. Therefore, we used 0.25, 0.5 and 1.0 μmol/L guaiacol for in vitro experiments and found that it suppressed early osteoclastogenesis in a dose‐dependent manner. In addition, it inhibited osteoclast function. The F‐actin ring is the structural basis of osteoclast function. RANKL and M‐CSF induce osteoclastogenesis, and M‐CSF triggers the proliferation and differentiation of pre‐osteoclasts.^28^, ^29^ Guaiacol did not affect the proliferation of BMMCs but inhibited osteoclastogenesis and osteoclast function in the early stages of differentiation. It is known that osteoblasts play an essential role in bone metabolism. So, the influence of guaiacol on adipogenesis and osteogenesis of BMSCs was detected in this study. However, guaiacol could not promote the osteogenesis and adipogenesis.^30^, ^31^ Our findings indicate that guaiacol inhibits osteoclastogenesis.

The binding of RANKL to RANK recruits TRAF6 and C‐Src.^29^ The RANK‐TRAF6 complex activates the NF‐κB and MAPK pathways, which involves phosphorylation of ERK, p38, JNK, IκB, p50, c‐fos and p65.^32^ The RANK‐C‐Src complex activates the AKT pathway, which involves phosphorylation of Akt^29^; this triggers Ca^2+^ oscillation^33^, ^34^ and the activation and nuclear translocation of NFATc1, an important osteoclastogenesis‐related transcription factor.^35^, ^36^ We found that guaiacol blocked interactions between RANK and TRAF6 and C‐Src, inhibiting the RANKL‐induced activation of the NF‐κB, AKT and MAPK pathways. Guaiacol did not decrease the transcription of RANK and c‐fms but suppressed RANKL‐induced Ca^2+^ oscillation. The expression of osteoclastogenesis‐related genes (MMP‐9, TRAP, cathepsin K and CTR^37^, ^38^) was inhibited by guaiacol in a dose‐dependent manner. MMP‐9 is associated with osteoclast function and contributes to absorption of bone matrix. Finally, the activation and nuclear translocation of NFATc1 were inhibited by guaiacol. Therefore, guaiacol inhibited osteoclastogenesis by inhibiting the RANKL‐induced activation of the NF‐κB, MAPK, AKT signalling pathways, Ca^2+^ oscillation and NFATc1 expression.

Postmenopausal osteoporosis is characterized by decreased bone mass and an imbalance of bone resorption and formation.^39^, ^40^ The enhanced osteoclastogenesis and osteoclast function caused by oestrogen withdrawal cause the pathologic changes in PMOP.^16^ To assess its effect on ovariectomy‐induced bone loss, we treated OVX mice with guaiacol.^41^ The maximum concentration of guaiacol usable in vivo was 125 mg/kg. H&E staining and micro‐CT showed that guaiacol significantly inhibited bone loss in OVX mice treated with guaiacol for 6 weeks. In addition, TRAP staining showed that guaiacol reduced the number of osteoclasts around the trabecula in OVX mice. Similarly, the ELISA results indicated that guaiacol decreased the serum levels of CTX‐1 and TRAcp5B, markers of bone resorption and the number of osteoclasts.

However, the target of guaiacol is unknown. We plan to design a series of compounds to block the target and thus inhibit osteoclastogenesis.

We extracted guaiacol from AS by 2D BMMCs/CMC/C18 column/TOFMS and found that it inhibited RANKL‐induced osteoclastogenesis and bone loss. Guaiacol blocked interactions between RANK and TRAF6 and C‐Src, and suppressed osteoclastogenesis by inhibiting the activation of the NF‐κB, MAPK, and AKT pathways and the Ca^2+^ signalling pathway. Collectively, guaiacol shows promise as a safe and effective therapeutic for bone disease. Through the subsequent structure modification of guaiacol, more derivatives with lower toxicity and better effect will be synthesized. The guaiacol will be used to treat the osteoclasts‐related pathological bone loss diseases in the future.

## CONFLICT OF INTERESTS

The authors confirm that there are no conflicts of interest.

## AUTHOR CONTRIBUTIONS

X. Zhi, X. Chen and J. Su designed the study. X. Zhi, C. Fang, H. Chen, Q. Zhou, Y. Wang, H. Jiang, YJ. Wang, Y. Gu, XF. Chen and J. Cui performed the experiments. Y. Hu, L. Cao, X. Li and W. Weng analysed the data. X. Chen and J. Su interpreted the data. X. Zhi, C. Fang and H. Chen wrote the manuscript.

## Supporting information

Figure S1

Figure S2

Figure S3

Figure S4

Figure S5

Figure S6

## Data Availability

Data used to support the findings of this study have been presented in the Supplementary Information.
