# Corrigendum

**DOI:** 10.1111/jcmm.16057

**Published:** 2021-01-17

In Zhi et al,^1^ the published article contains errors in Figures 6 and 7. The correct figures are shown below. The authors confirm all results and conclusions of this article remain unchanged.

**Figure 6:**
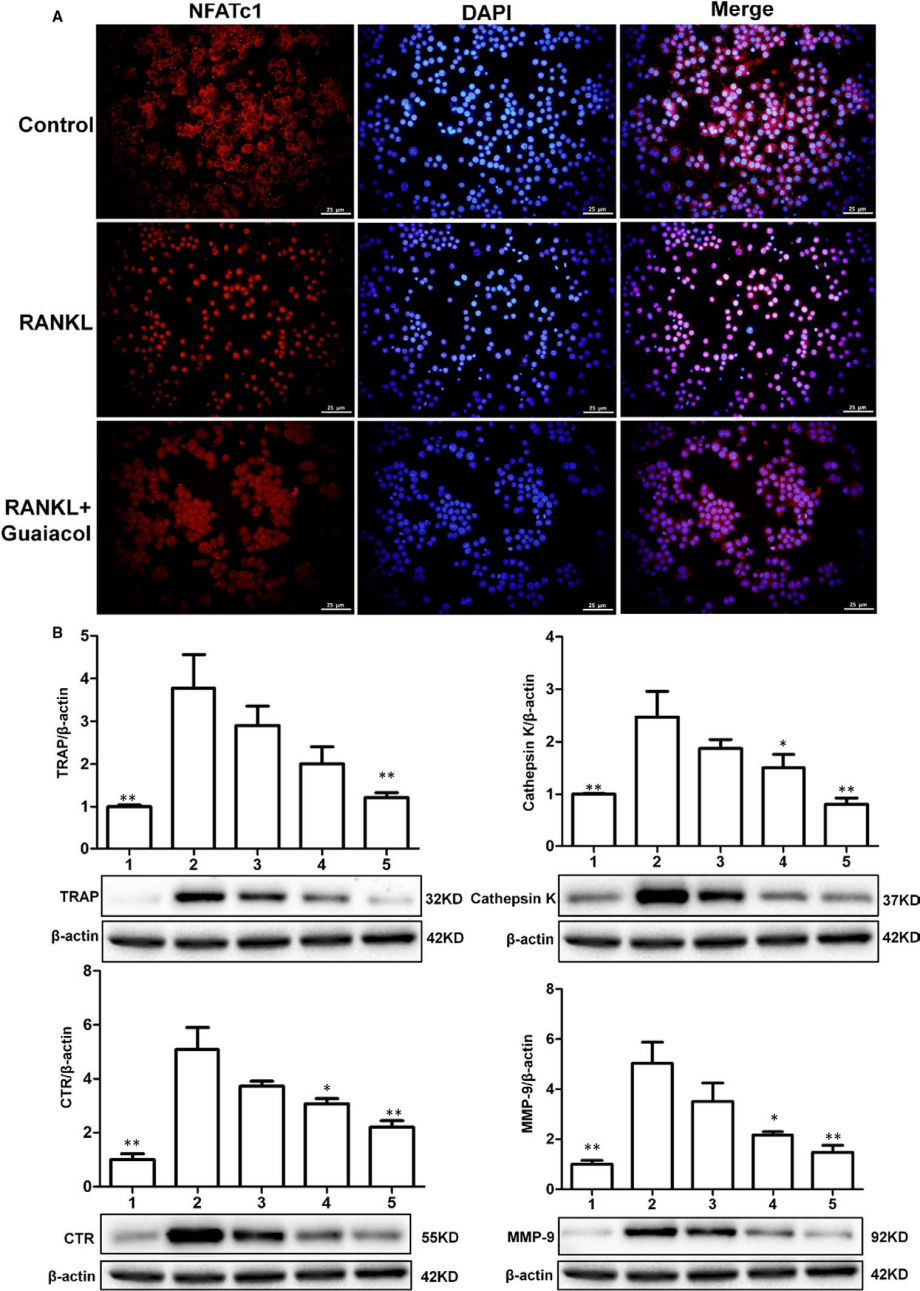
Guaiacol suppressed the expression of NFATc1 and osteoclastogenesis‐related genes. A, RANKL induced the nuclear translocation of NFATc1 during osteoclastogenesis, while guaiacol inhibited NFATc1 expression. B, Western blotting of cathepsin K, CTR, MMP‐9 and TRAP, with β‐actin as a reference. 1. RAW264.7 cells; 2. RAW264.7 cells induced with M‐CSF (30 ng/mL), RANKL (50 ng/mL) and PBS; 3. RAW264.7 cells induced with M‐CSF (30 ng/mL) and RANKL (50 ng/mL) and treated with 0.25 μmol/L guaiacol; 4. RAW264.7 cells induced with M‐CSF (30 ng/mL) and RANKL (50 ng/mL) and treated with 0.5 μmol/L guaiacol; 5. RAW264.7 cells induced with M‐CSF (30 ng/mL) and RANKL (50 ng/mL) and treated with 1 μmol/L guaiacol. *P < .05, **P < .01

**Figure 7:**
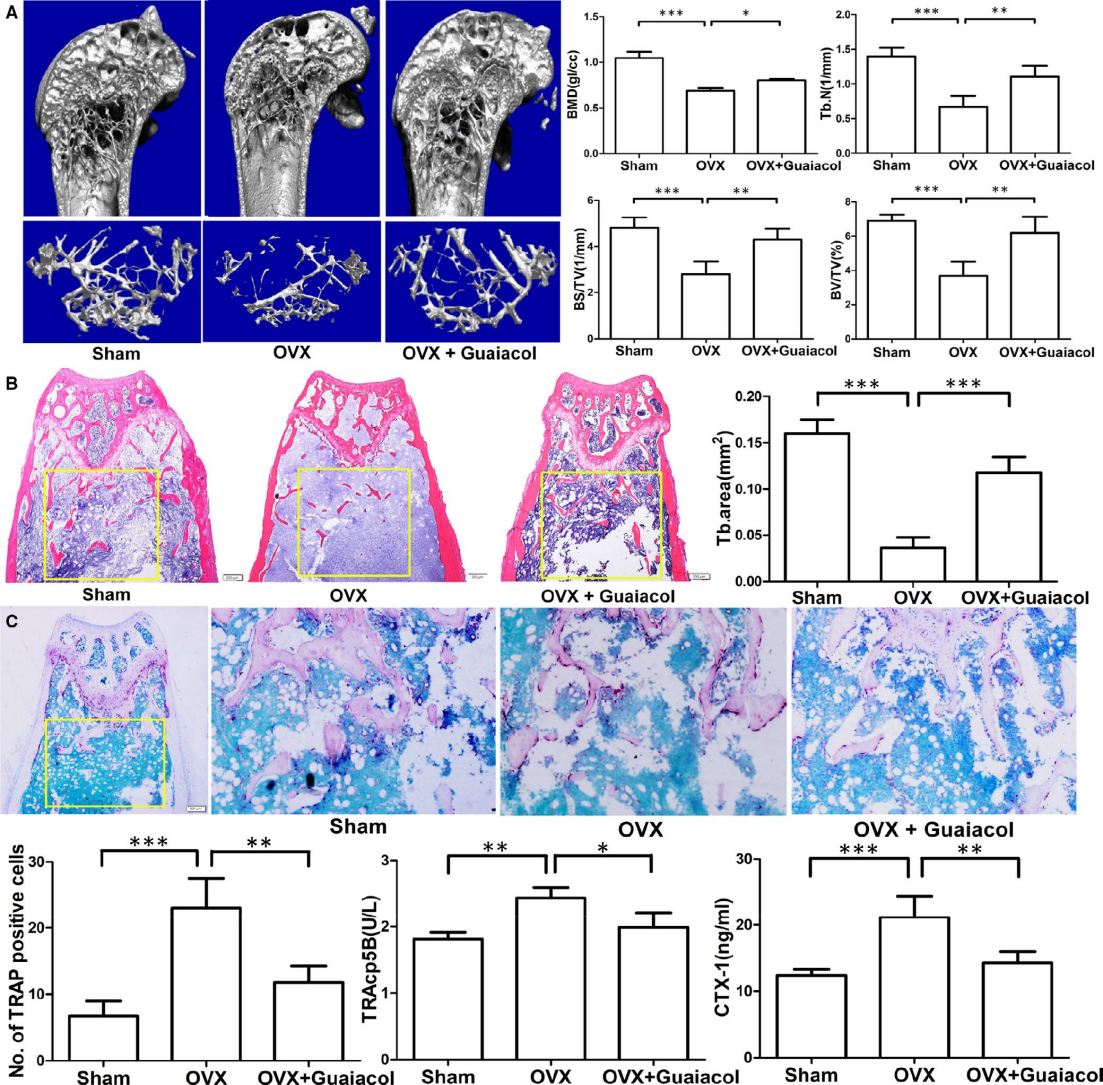
Guaiacol inhibited bone loss in OVX mice. A, Micro‐CT analyses of the distal femur of mice in the sham, OVX and OVX + guaiacol groups. B, H&E staining of sections of the distal femur and trabecular area at 6 wk after treatment. C, TRAP‐stained sections of the distal femur and number of TRAP‐positive cells in mice in the sham, OVX and OVX + guaiacol groups. D, Level of TRAcp5B and CTX‐1 as determined by ELISA. *P < .05, **P < .01, ***P < .001
