# Supplementary figures and images for: Guaiacol suppresses osteoclastogenesis by blocking interactions of RANK with TRAF6 and C‐Src and inhibiting NF‐κB, MAPK and AKT pathways
Source: J Cell Mol Med. 2020 Mar 17;24(9):5122–34. doi: 10.1111/jcmm.15153 (PMC7205840; doi:10.1111/jcmm.15153)

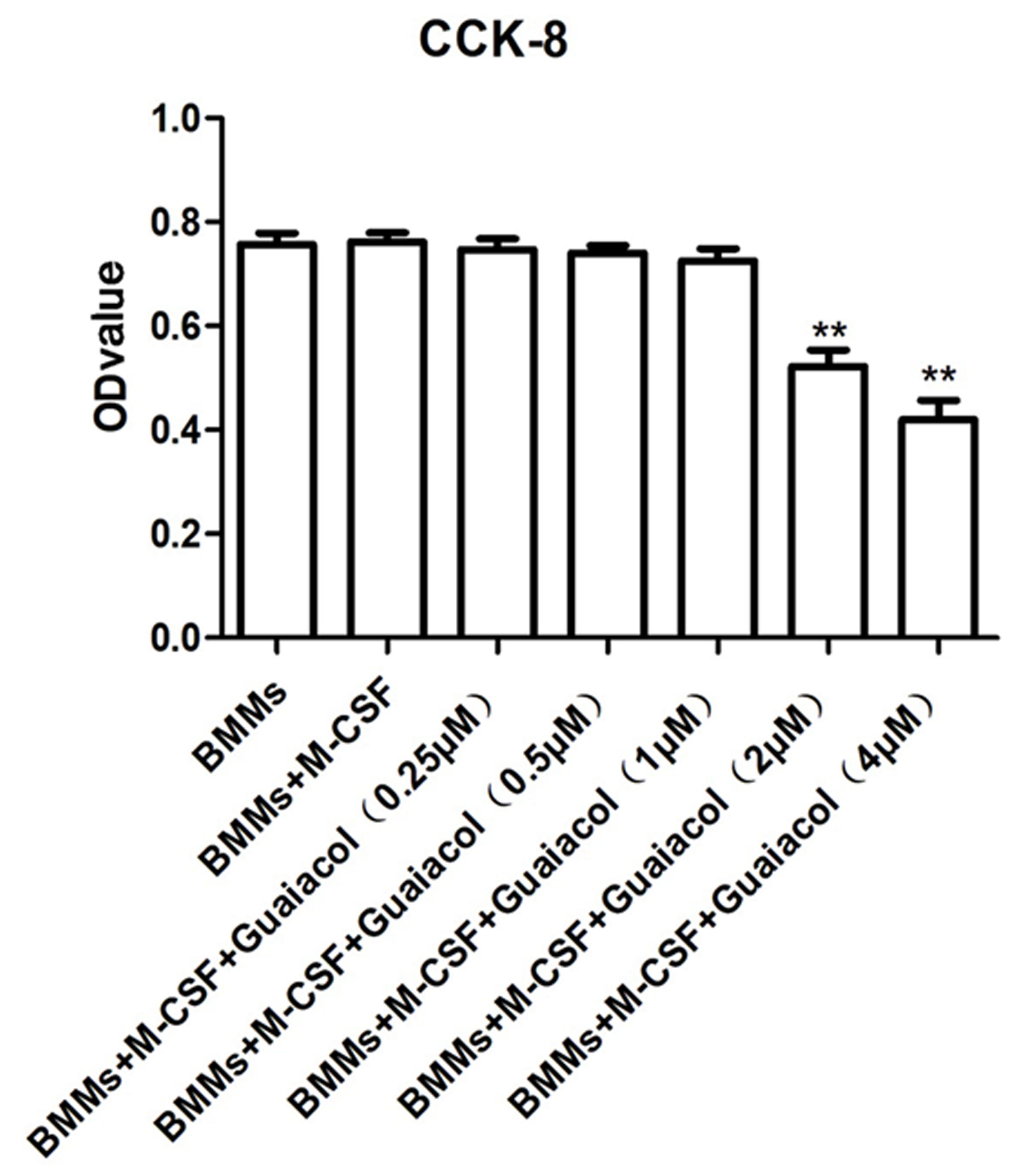

Supplement: Supplementary file 1 — Figure S1 [file JCMM-24-5122-s001.tif]

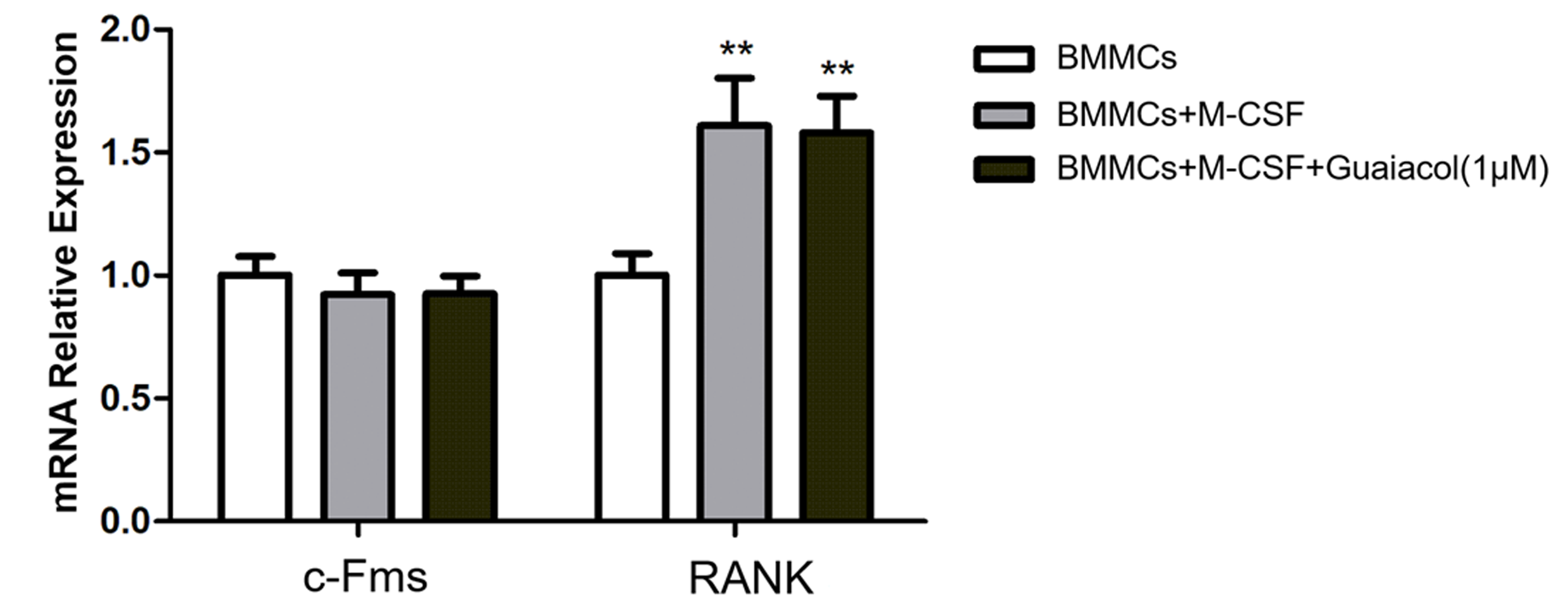

Supplement: Supplementary file 2 — Figure S2 [file JCMM-24-5122-s002.tif]

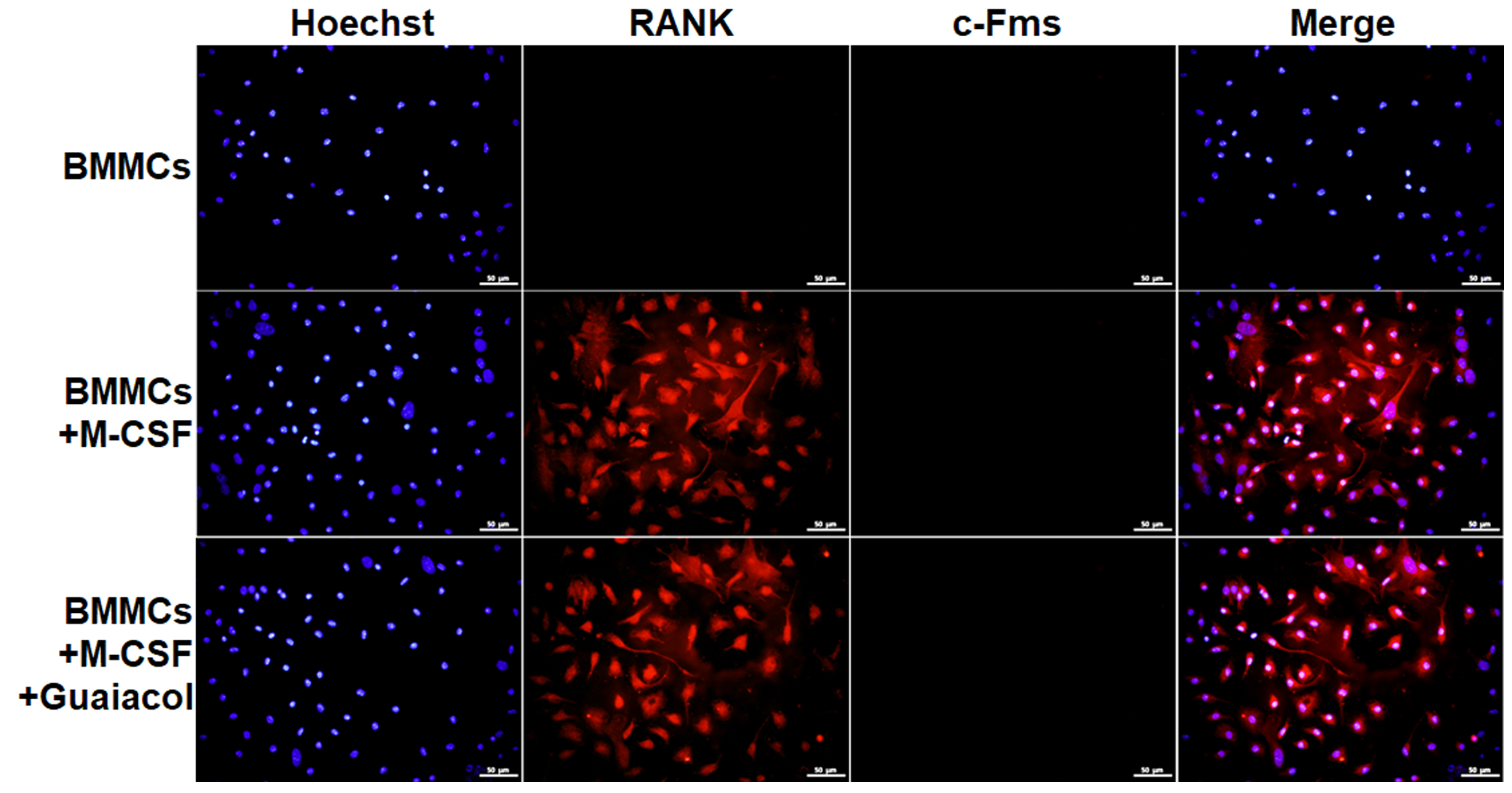

Supplement: Supplementary file 3 — Figure S3 [file JCMM-24-5122-s003.tiff]

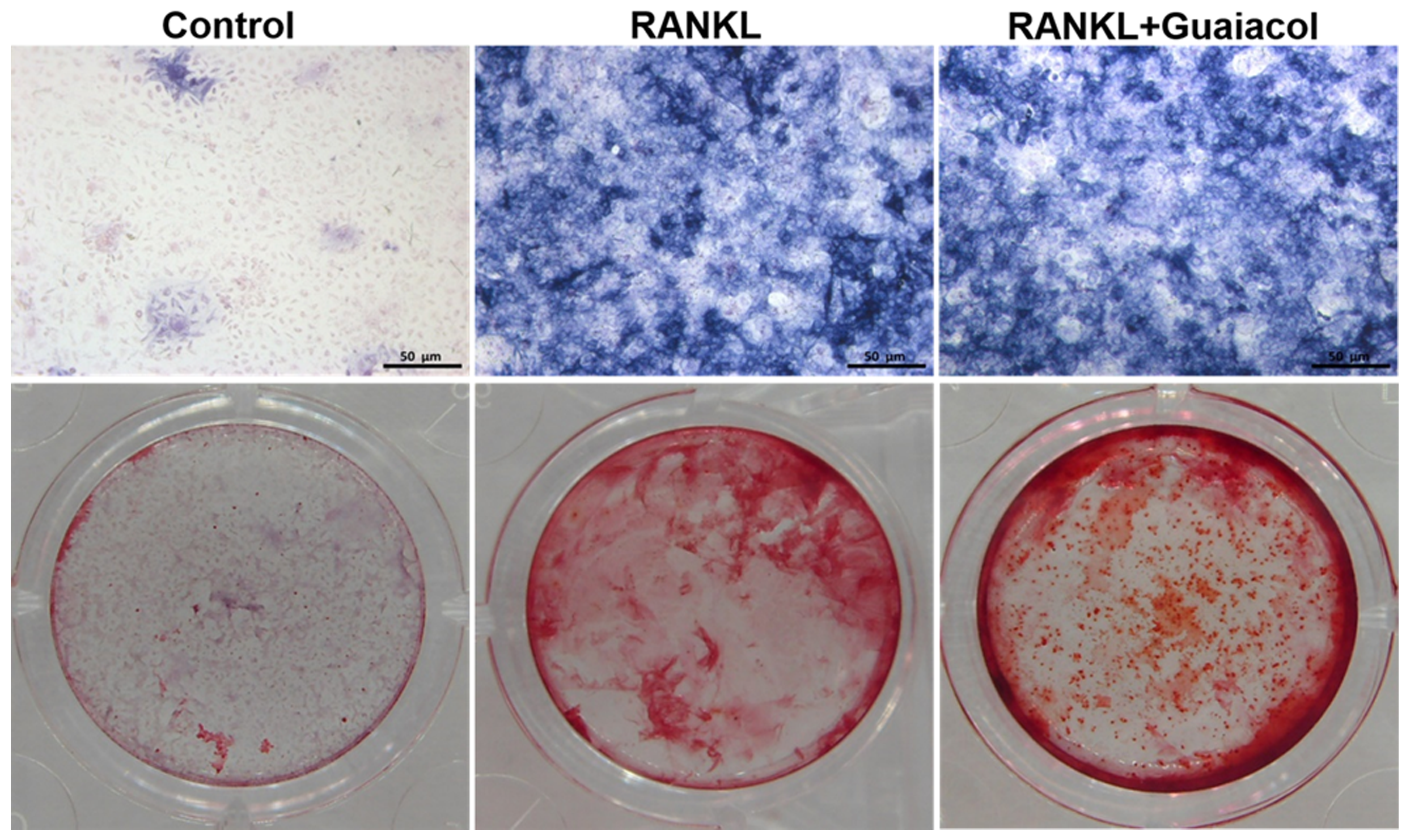

Supplement: Supplementary file 4 — Figure S4 [file JCMM-24-5122-s004.tif]

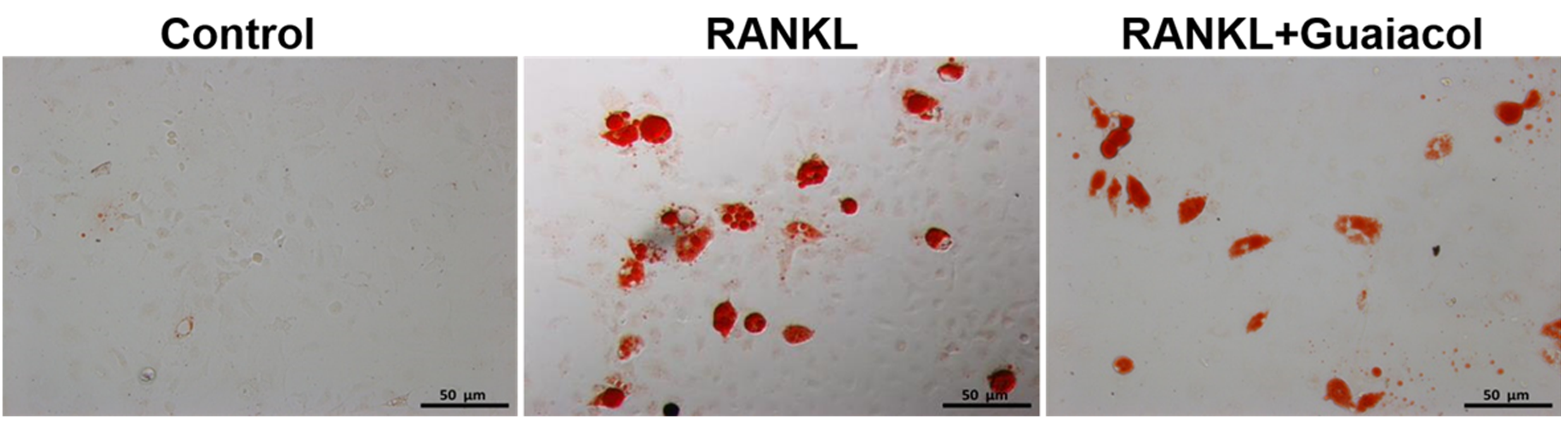

Supplement: Supplementary file 5 — Figure S5 [file JCMM-24-5122-s005.tif]

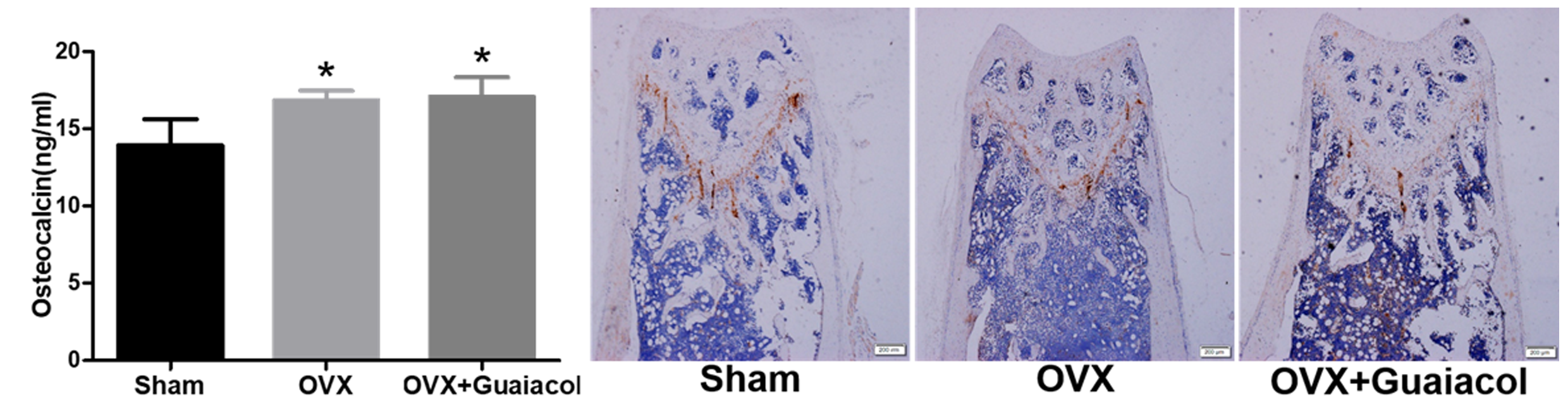

Supplement: Supplementary file 6 — Figure S6 [file JCMM-24-5122-s006.tif]
